# Differences in circulating obesity-related microRNAs in Austrian and Japanese men: A two-country cohort analysis

**DOI:** 10.1016/j.metop.2022.100206

**Published:** 2022-08-17

**Authors:** Ichiro Wakabayashi, Yoko Sotoda, Klaus Groschner, Peter P. Rainer, Harald Sourij

**Affiliations:** a Department of Environmental and Preventive Medicine, School of Medicine, Hyogo Medical University, Nishinomiya, Hyogo, 663-8501, Japan; b Department of Cardiovascular Surgery, Yamagata Saisei Hospital, Yamagata, 990-8545, Japan; c Gottfried Schatz Research Center for Cell Signaling, Metabolism and Aging, Medical University of Graz, Neue Stiftingtalstrasse 6/D04, 8010, Graz, Austria; d Division of Cardiology, Medical University of Graz, Auenbruggerplatz 15, 8036, Graz, Austria; e Division of Endocrinology and Diabetology, Medical University of Graz, Auenbruggerplatz 15, 8036, Graz, Austria

**Keywords:** Canonical pathway, Ethnic difference, Leptin, MicroRNA, Obesity

## Abstract

**Background:**

The prevalence of obesity is higher in Western countries than in East Asian countries. It remains unknown whether microRNAs (miRNAs) are involved in the pathogenesis of the ethnic difference in obesity. The purpose of this study was to determine whether expression levels of circulating obesity-associated miRNAs are different in Europeans and Asians.

**Methods:**

The subjects were middle-aged healthy male Austrians (n = 20, mean age of 49.9 years) and Japanese (n = 20, mean age of 48.7 years). Total miRNAs in serum from each subject were analyzed using the 3D-Gene miRNA Oligo chip. miRNAs that showed significant differences between the Austrian and Japanese groups were uploaded into Ingenuity Pathway Analysis (IPA).

**Results:**

Among 16 miRNAs that were revealed to be associated with obesity in previous studies and showed expression levels that were high enough for a reasonable comparison, serum levels of 3 miRNAs displayed significant differences between the Austrian and Japanese groups: miR-125b-1-3p was significantly lower with a fold change of −2.94 and miR-20a-5p and miR-486–5p were significantly higher with fold changes of 1.73 and 2.38, respectively, in Austrians than in Japanese. In IPA including all 392 miRNAs that showed significant differences between Austrians and Japanese, three canonical pathways including leptin signaling in obesity, adipogenesis pathway and white adipose tissue browning pathway were identified as enriched pathways.

**Conclusions:**

miRNAs are thought to be involved in the ethnic difference in the prevalence of obesity, which may in part be caused by different expression levels of miR-125b-1-3p, miR-20a-5p and miR-486–5p.

## Highlights

• Ethnic difference in circulating obesity-related microRNAs (miRs) were investigated.
• Levels of 2565 miRs in blood were compared between Austrians and Japanese.
• miR-125b-1-3p, −20a-5p and -486–5p levels were significantly different between them.
• Significant ethnic differences were found in blood levels of 392 miRs.
• These miRs target the molecules comprising obesity-related three canonical pathways.

## 1 Introduction

Obesity is a common worldwide health problem and causes an increased risk of various diseases including cardiovascular disease, non-insulin-dependent diabetes mellitus, obstructive pulmonary disease, arthritis and cancer [1]. The basic cause of obesity is an imbalanced energy homeostasis with an excess of calories consumed at a paucity of calories expended, and this imbalance is propelled by a variety of factors related to overeating, low energy expenditure and insufficient physical activity. There is a large heterogeneity in the prevalence of obesity, which is in part caused by individual factors including socio-economic and ethnic differences [2].

Heritability is well recognized as an important cause of obesity. The intrapair correlation coefficients of the values for body mass index (BMI) of identical twins reared apart were reported to be 0.70 for men and 0.66 for women [3]. The heritability of BMI and waist circumference was estimated to be 77% for both in a previous study on 5092 identical and non-identical twin pairs aged 8–11 years [4]. By genome-wide association studies (GWAS), a large number of gene variants were identified to be associated with the prevalence of obesity defined as high BMI; however, those gene variants account for only a small percentage of individual variation of obesity [5].

Genetic susceptibility to obesity involves epigenetic modifications including DNA methylation and histone modification, which are influenced by age and environmental factors such as diet and physical activity [6]. microRNAs (miRNAs), non-coding single-strand RNAs consisting of 20–25 bases, also play a role in epigenetic modifications of obesity-associated genes [7,8]. Circulating miRNAs are also promising biomarkers for detection of various diseases, and there has been an accumulation of information on circulating miRNAs that are different in individuals with and without obesity [[9], [10], [11]]. Although ethnic differences are related to the prevalence of obesity [12], it is not known whether and how miRNAs are involved in the ethnic difference in obesity.

**Fig. 1:**
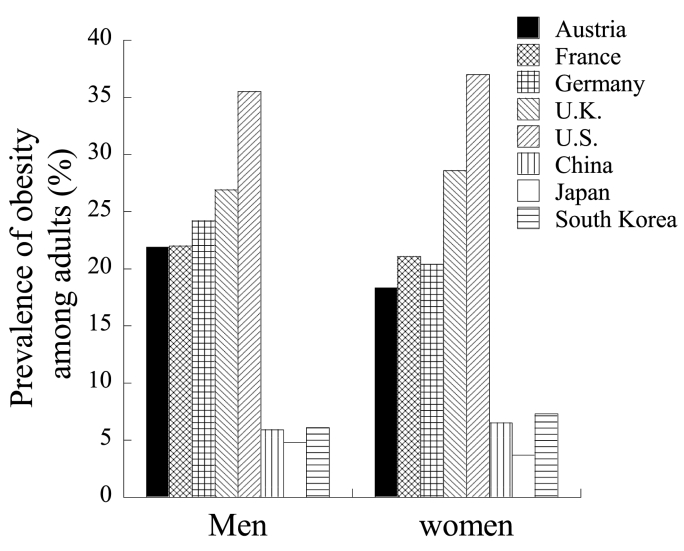
Comparison of the prevalences of obesity in adult men and women in Western and East Asian countries. Age-standardized estimates of prevalence are shown (12).

The purpose of this study was therefore to investigate the ethnic difference in obesity-related miRNAs and to explore the possibility of miRNAs as a cause of ethnic difference in the prevalence of obesity. As shown in Fig. 1, there is a great difference in the prevalences of obesity, defined as BMI of 30 kg/m^2^ or higher, in Western countries and East Asian countries [12]. The highest prevalence of obesity is in the U.S., which is a multiethnic country. In Western countries, the cutoffs for overweight and obesity based on BMI are 25 and 30 kg/m^2^, respectively. When this cutoff is used, the prevalence of obesity (BMI of 30 kg/m^2^ or higher) is only 2–4% in East Asian countries. From the viewpoint of prevention of type 2 diabetes and hypertension, lower (stricter) cutoffs such as 23 kg/m^2^ for overweight and 25 kg/m^2^ for obesity are recommended in East Asian countries [13]. According to WHO global estimates, 39% and 13% of adults aged 18 years and over were overweight and obese, respectively, in the world in 2016 [14]. In the U.S., prevalences of obesity (BMI of 30 kg/m^2^ or higher) and severe obesity (BMI of 40 kg/m^2^ or higher) from 2017 to March 2020 were 41.9% and 9.2%, respectively [15]. In this study, we compared circulating obesity-related miRNA levels in healthy Austrian and Japanese men as representatives of individuals in Western countries and East Asian countries. Prevalences of obesity among adults (both sexes) were 20.1% in Austria and 4.3% in Japan [12].

## 2 Methods

### 2.1 Participants

The participants in this study were healthy male Austrians (n = 20) and Japanese (n = 20). All of the Austrian participants were Caucasians, and all of the Japanese participants were originally from Japan. We tried to enroll representative healthy middle-aged Austrian and Japanese men into this study. Those receiving any medication therapy and those with histories of known inflammatory, metabolic and cardiovascular disorders or malignancy were excluded from the participants of this study. Smokers were also excluded from the participants. The Japanese participants were male healthy volunteers who were working in different districts including three cities, Nishinomiya, Sasayama or Yamagata, in Japan. The Austrian participants were recruited at the Medical University of Graz, Graz, Austria. The protocol of this study was approved by the Hyogo College of Medicine Ethics Committee (No. 3036 in 2018) and the Medical University of Graz Ethics Committee (27–166 ex 14/15). Written informed consent was provided by all of the participants. All methods were performed in accordance with the relevant guidelines and regulations.

### 2.2 Blood sample collection

Blood was collected from each participant after overnight fasting, and serum was separated. Serum samples were kept frozen at −80°C until analyses of miRNAs and measurements of leptin and adiponectin as described below.

### 2.3 Measurements of variables related to obesity

Height, body weight and waist circumference were measured, and BMI and waist-to-height ratio were calculated as weight in kilograms divided by the square of height in meters and waist circumference in cm divided by height in cm, respectively. Serum leptin and adiponectin concentrations were measured by enzyme immunoassays using commercial kits, Leptin (Sandwich EIA) Human (EIA2395R) from DRG and ADIPONECTIN, ELISA, HUMAN (BVL-RD195023100-1) from BioVendor, respectively.

### 2.4 RNA extraction and miRNA expression profiling

RNA was extracted from a serum sample (300 μl) using 3D-Gene RNA extraction reagent from a liquid sample kit (Toray Industries Inc., Kamakura, Japan) according to the manufacturer's instructions as described previously [16]. miRNA expression was analyzed using the 3D-Gene miRNA Oligo chip (TRT-XR520, Toray) and 3D-Gene miRNA labeling kit (TRT-XE211, Toray) as described previously [17]. Briefly, half volumes of labeled RNAs were hybridized onto a 3D-Gene miRNA Oligo chip (Toray), which was designed to detect sequences of multiple miRNAs. The annotations and oligonucleotide sequences of the probes conformed to those in miRBase release 21, an miRNA database (http://microrna.sanger.ac.uk/sequences/). After stringent washes, fluorescent signals were scanned with a 3D-Gene Scanner (Toray) and analyzed using 3D-Gene Extraction software (Toray). miRNA expression was normalized as follows: The raw data of each spot were substituted with a mean intensity of the background signal determined by signal intensities of all blank spots with signal intensity of the top and bottom 5% (out of 95% confidence intervals) being removed. Measurements of spots were considered to be valid when the signal intensities were greater than 2 standard deviations of the background signal intensity. The signal intensities of the valid spots were compared and the relative expression level of a given miRNA was calculated. Global normalization of the data was performed for each array, such that the median of the signal intensity was adjusted to 25. Intensity levels of 10 or higher were regarded to be high enough for analysis of comparison between the two country groups. Each miRNA level was compared after log2 transformation between the groups. Fold change in the mean value of each miRNA intensity of Austrian versus Japanese subjects was calculated as the ratio of anti-log2 values of each mean of log2-transformed data.

### 2.5 Selection of obesity-associated miRNAs

A total of 2565 miRNAs in serum were measured by using the miRNA Oligo chip. The intensities of 1744 miRNA were too low for reasonable quantitative comparison. Consequently, 821 miRNAs were available for comparison between the Austrian and Japanese groups. Among these 821 miRNAs, 392 miRNAs showed significant differences in the Austrian and Japanese groups.

From previous reports [[18], [19], [20], [21], [22], [23], [24], [25], [26], [27], [28]], we further chose 46 miRNAs that have been reported to be associated with obesity as shown in Table 1. Among these miRNAs, 16 miRNAs (miR-103a-3p, miR-125b-1-3p, miR-15a-5p, miR-17–5p, miR-197–3p, miR-20a-5p, miR-221–3p, miR-223–3p, miR-23a-3p, miR-23b-3p, miR-320a, miR-370–3p, miR-423-5p, miR-486–3p, miR-486–5p, miR-758–5p) that showed serum levels high enough for comparison were used for further analysis.

**Table 1:** Relationships of circulating miRNAs with overweight and obesity in previous studies.

miRNAs	Source	Condition	Alteration	References
**miR-132, 17-5p**	whole blood	obese	down	[18]
**miR-103, 155, 21, 27b**	whole blood	obese	down	[19]
**miR-125b, 130b, 15a, 221, 423-5p, 520c-3p, 532-5p** **miR-140-5p, 142-3p, 222**	plasma	severe obese	down up	[20]
**miR-138, 376a, 503** **miR-15b**	serum	obese	down up	[21]
**miR-221, 28-3p** **miR-130b, 142-3p, 423-5p, 486-3p, 486-5p**	plasma	obese (prepubertal children)	down up	[22]
**miR-143, 335, 758** **miR-27, 370, 378**	serum	obese (children & adolescents)	down up	[23]
**miR-223**	plasma	overweight, obese	down	[24]
**miR-206** **miR-2355-5p, 31-5p**	plasma	overweight & obese (prepubertal children)	down up	[25]
**miR-130a-3p, 197-3p, 221-3p, 23-3p, 27a-3p, 320a**	serum	obese	down	[26]
**miR-146a, 15a, 423-5p, 520c-3p, 532-5p** **miR-130, 140-5p, 142-3p, 143, 222**	plasma	overweight, obese (adolescents)	down up	[27]
**miR-197** **miR-146a, 146b, 15b, 20a, 222, 26b, 486**	serum	obese	down up	[28]

### 2.6 Bioinformatics pathway analysis

The resultant miRNAs expressed in serum that showed significant differences in the Austrian and Japanese groups were analyzed using Ingenuity Pathway Analysis (IPA) (Ingenuity Pathway Analysis: QIAGEN Inc. https://www.qiagenbioinformatics.com/products/ingenuitypathway-analysis) [29]. Putative miRNA targets were found using Ingenuity miRNA target filter for experimentally validated and putative predicted targets (high confidence level) through in silico analysis [30]. After linking the miRNAs with their mRNA target genes, mRNA target genes that were not significantly different between the countries were filtered out. The IPA miRNA-mRNA target link module derives information from TargetScan, a database containing miRNAs and their predicted target genes, along with prediction scores and experimental conformation from the literature [31].

### 2.7 Statistical analysis

Continuous variables are summarized as means with standard deviations and were compared with the use of Welch's corrected *t*-test. For each of the tested miRNAs, on the basis of the observed distribution of *p* values, we estimated the positive false discovery rate (*q* value) according to the method of Storey et al. [32]. Associations among the miRNAs with significant *q* values were explored with the use of Pearson's correlation coefficient. The intensity of each miRNA was transformed with the base-2 logarithm, that is, binary logarithm. We performed univariable and multivariable linear regression analyses in order to know the relationships among the three miRNAs (miR-125b-1-3p, -20a-5p and -486–5p) that were previously reported to be associated with obesity and were significantly different in Austrian and Japanese men in the present study. The intensity of each miRNA was transformed with the base-2 logarithm for normal distribution. Pearson's correlation coefficient and standardized partial regression coefficient (β) were calculated in the univariable and multivariable analyses, respectively. In multivariable linear regression analysis for the relationship between two miRNAs out of miR-125b-1-3p, -20a-5p and -486–5p, adjustment was performed by using levels of the miRNA (as the other explanatory variable for adjustment) other than the above two paired miRNAs (an explanatory variable and an independent variable). Thus, only two variables of miRNA were used as the explanatory variables, and no other variables were used for adjustment in multivariable linear regression analysis. All *p* values were two-sided, and *p* values less than a significance level of 0.05 were considered statistically significant. All *q* values were two-sided, with statistical significance determined by a false discovery rate of less than 0.05. Data were analyzed with the use of SPSS version 25.0 Armonk, NY, USA.

## 3 Results

### 3.1 Comparison of adiposity-related variables in Austrian and Japanese men

Table 2 shows adiposity-related variables of the Austrian and Japanese subjects. Height, weight and waist circumference in the Austrian group were significantly larger than those in the Japanese group. Adiposity indices, BMI and waist-to-height ratio, were significantly higher in the Austrian group than in the Japanese group. Leptin and adiponectin levels were not significantly different in the Austrian and Japanese groups.

**Table 2:** Comparison of adiposity-related variables in Austrian and Japanese subjects.

Variables	Austrian men	Japanese men	*p* values
**Number**	20	20	–
**Age (years)**	49.9 ± 6.3	48.7 ± 6.4	0.551
**Height (cm)**	180.3 ± 5.7**	173.2 ± 4.5	<0.001
**Weight (kg)**	83.7 ± 9.1**	70.0 ± 10.6	<0.001
**Waist circumference (cm)**	94.3 ± 7.6**	82.6 ± 9.6	<0.001
**Body mass index (kg/m**^**2**^**)**	25.7 ± 1.9**	23.3 ± 3.1	0.006
**Waist-to**-**height ratio**	0.523 ± 0.039**	0.477 ± 0.053	0.003
**Leptin (ng/ml)**	2.21 ± 1.43	1.98 ± 1.72	0.649
**Adiponectin (μg/ml)**	8.59 ± 2.23	8.42 ± 3.34	0.854

Shown are means with standard deviations of each variable. Asterisks denote significant differences from Japanese (**, *p* < 0.01).

### 3.2 Comparison of obesity-related miRNA levels in Austrian and Japanese men

Table 3 shows the results of a comparison of obesity-related miRNA levels in the Austrian and Japanese groups. There were 16 miRNAs that showed levels high enough for comparison and were reported to be associated with obesity. Among those miRNAs, the levels of miR-103a-3p, -15a-5p, -17–5p, -20a-5p, -320a, -423–5p, -486–5p and -758–5p were significantly higher in the Austrian group than in the Japanese group, while the levels of miR-125b-1-3p and -370–3p were significantly lower in the Austrian group than in the Japanese group. The levels of miR-197–3p, -221–3p, -223–3p, -23a-3p, -23b-3p and -486–3p were not significantly different between the two groups. Relatively high values (>1.5) of fold change were found in miR-125b-1-3p, -15a-5p and -486–5p. Also shown in Table 3 are reported changes (up or down) in miRNA levels of individuals with obesity compared with those without obesity: miR-103, -125b, -15a, -17–5p, -197–3p, -221–3p, -223, -23–3p, -320a, -423–5p and -758 levels were reported to be lower in individuals with obesity than in those without obesity [19,20,[22], [23], [24],[26], [27], [28]], while miR-20a, -370, -486–3p and -486–5p levels were reported to be higher in obese individuals than in non-obese individuals [22,28]. Therefore, miR-125b-1-3p, -20a-5p and -486–5p were miRNAs that were reported to be related to obesity and were significantly different in Austrian and Japanese men in the present study.

**Table 3:** Comparison of obesity-related miRNAs in Austrian and Japanese subjects.

miRNA	Intensity^#^	**Fold change**^##^	*q* value	**Change**^###^	References
**103a-3p**	5.63 ± 0.78** 4.81 ± 0.55	1.76	0.00068	down	[19]
**125b-1-3p**	5.72 ± 0.99** 7.27 ± 0.45	−2.94	8.4E-07	down	[20]
**15a-5p**	5.15 ± 1.07** 3.75 ± 0.72	2.62	5.3E-05	down	[20,27]
**17-5p**	5.96 ± 0.90** 5.24 ± 0.60	1.63	0.00542	down	[18]
**197-3p**	4.74 ± 0.38 4.67 ± 0.28	1.04	0.25370	down	[26,28]
**20a-5p**	5.27 ± 1.16* 4.47 ± 0.75	1.73	0.01737	up	[28]
**221-3p**	4.95 ± 0.60 4.95 ± 0.67	1.00	0.43103	down	[20,22,26]
**223-3p**	6.63 ± 0.81 6.97 ± 0.76	−1.27	0.13817	down	[24]
**23a-3p**	6.02 ± 0.61 6.08 ± 0.51	−1.04	0.37181	down	[26]
**23b-3p**	6.00 ± 0.66 5.96 ± 0.55	1.02	0.40312	down	[26]
**320a**	5.99 ± 0.61** 5.59 ± 0.32	1.31	0.00273	down	[26]
**370-3p**	6.23 ± 0.42** 6.49 ± 0.16	−1.20	0.00078	up	[23]
**423-5p**	8.13 ± 0.52** 7.65 ± 0.24	1.39	0.00018	down	[20,22,27]
**486-3p**	8.72 ± 0.38 8.85 ± 0.24	−1.10	0.09862	up	[22,28]
**486-5p**	8.65 ± 1.05** 7.40 ± 0.55	2.38	3.1E-05	up	[22,28]
**758-5p**	3.94 ± 0.34* 3.68 ± 0.31	1.20	0.01191	down	[23]

^**#**^Means with standard deviations of log_2_-transformed intensities of each miRNA in Austrians (upper) and Japanese (lower). ^##^Fold change in each miRNA intensity of Austrians versus Japanese. ^###^Reported change (up or down) in circulating miRNA levels of individuals with obesity compared with those without obesity. Asterisks denote significant differences from Japanese (*, *q* < 0.05; **, *q* < 0.01).

### 3.3 Relationships between obesity-related miRNAs

**Fig. 2:**
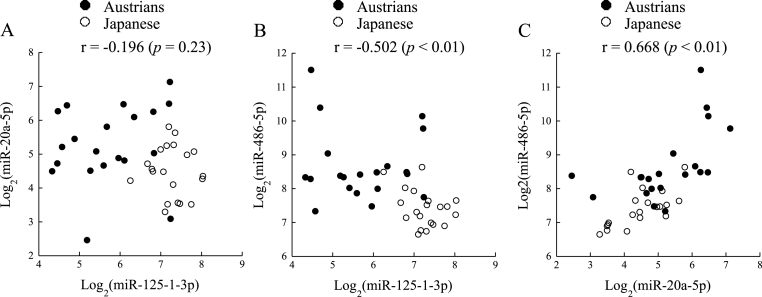
Correlations between miR-125-1-3p and miR-20a-5p levels (**A**), between miR-125-1-3p and miR-486–5p levels (**B**), and between miR-20a-5p and miR-486–5p levels (**C**). Closed circle, Austrians; open circle, Japanese. Pearson's correlation coefficients are shown.

Fig. 2 shows scatter plots of the correlations between obesity-related miRNAs. There was a significant positive correlation between miR-20a-5p and miR-486–5p levels and there was a significant inverse correlation between miR-125-1-3p and miR-486–5p levels. No significant correlation was found between miR-125-1-3p and miR-20a-5p levels. These relationships were not confounded by adjustment for each miRNA level other than paired miRNAs in multivariable linear regression analysis (standardized partial regression coefficient (β): between miR-125-1-3p and miR-20a-5p levels, 0.250 (*p* = 0.19); between miR-125-1-3p and miR-486–5p levels, −0.669 (*p* < 0.01); between miR-20a-5p and miR-486–5p levels, 0.761 (*p* < 0.01).

### 3.4 Obesity-associated pathway predicted to involve the miRNAs that were found significantly different in Austrian and Japanese men

**Fig. 3:**
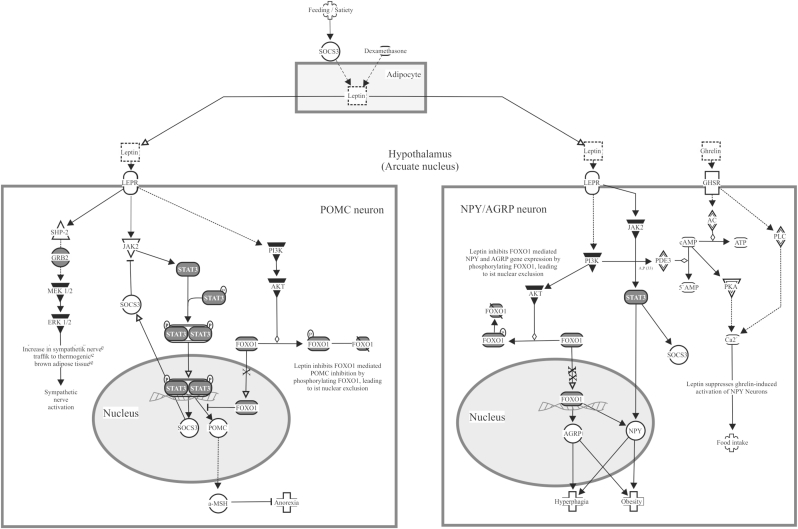
Overlap of identified miRNA targets with pathways related to the leptin signal in obesity. Highlighted are the molecules that showed significant associations in Ingenuity Pathway Analysis with the 392 miRNAs found to be significantly different between Austrian and Japanese subjects in this study.

**Fig. 4:**
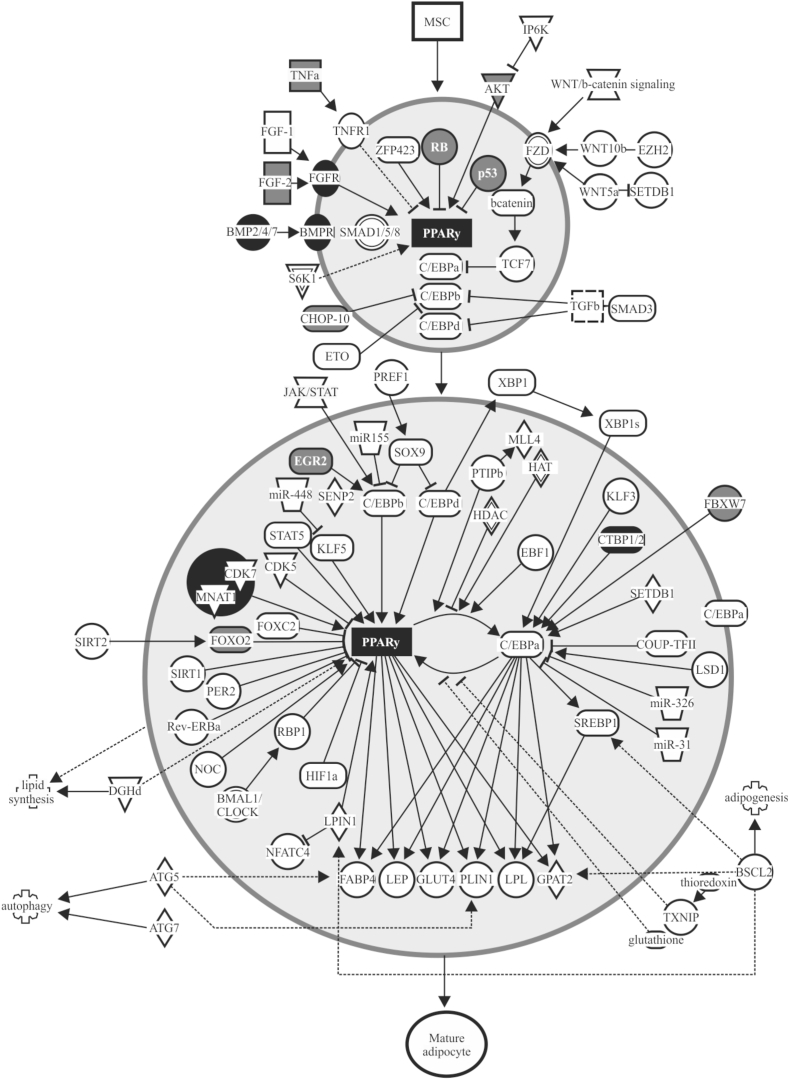
Overlap of identified miRNA targets with the adipogenesis pathway. Highlighted are the molecules that showed significant associations in Ingenuity Pathway Analysis with the 392 miRNAs found to be significantly different between Austrian and Japanese subjects in this study.

**Fig. 5:**
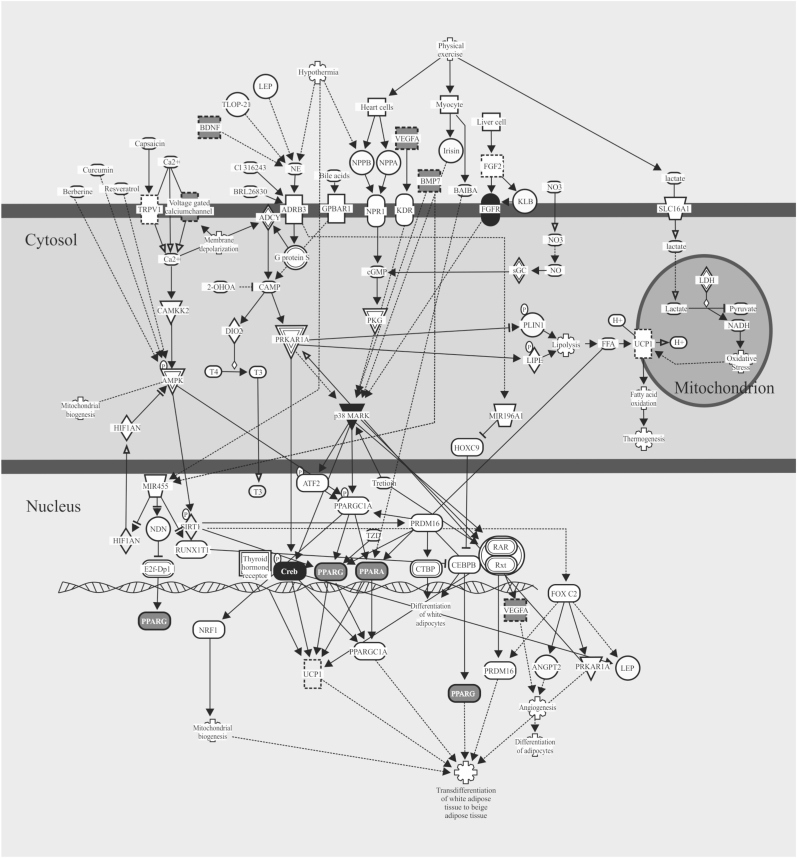
Overlap of identified miRNA targets with the white adipose tissue browning pathway. Highlighted are the molecules that showed significant associations in Ingenuity Pathway Analysis with the 392 miRNAs found to be significantly different between Austrian and Japanese subjects in this study.

IPA was performed using all of the miRNAs (n = 392) that showed significant differences in microarray expression analysis. Using a *p*-value filter for pathway significance of *p* < 0.05, 366 canonical pathways were identified as enriched pathways in the dataset of the miRNAs showing significant differences in the Austrian and Japanese groups. These canonical pathways include leptin signal in obesity (10 molecules in our dataset/76 molecules in the pathway [ratio: 0.132]; pathway *p* value = 1.79E-05 [Fig. 3]), adipogenesis pathway (16 molecules in our dataset/135 molecules in the pathway [ratio: 0.119]; pathway *p* value = 2.52E-07 [Fig. 4]), and white adipose tissue browning pathway (12 molecules in our dataset/138 molecules in the pathway [ratio: 0.087]; pathway *p* value = 1.73E-04 [Fig. 5]).

## 4 Discussion

The expression levels of 16 circulating miRNAs, which were reported as increased or decreased in obese individuals, were compared in the Austrian and Japanese subjects: miR-125b-1-3p was lower and miR-20a-5p and miR-486–5p were higher in the Austrian group than in the Japanese group and were thus suggested to be associated with the ethnic difference in the prevalence of obesity. This study is, to the best of our knowledge, the first study showing an ethnic difference in obesity-related miRNAs. On the other hand, among the 16 miRNAs tested, miR-221–3p, -223–3p and -486–3p levels were not different in the two groups and miR-103a-3p and -423–5p levels were significantly higher in the Austrian group than in the Japanese group, and this direction of changes in miRNAs was opposite to that shown in previous studies on obesity-associated miRNAs [19,20,22,27].

By using IPA, we further explored the canonical pathways that were targeted by all 392 miRNAs displaying significant differences in the Austrian and Japanese subjects. As a result, three obesity-related pathways, including leptin signaling in obesity, adipogenesis pathway, and white adipose tissue browning pathway, were found associated with the above 392 miRNAs. These miRNAs were suspected to target the molecules including STAT3, PI3K, AKT and FOXO1 in leptin signal in obesity, PPARγ and FOXO1 in adipogenesis pathway, and p38MAPK, PPARγ and PPARα in white adipose tissue browning pathway (Fig. 3, Fig. 4, Fig. 5). In the leptin signaling, leptin produced in adipocytes stimulates its receptors in POMC neurons and NPY/AGRP neurons in the hypothalamus and regulates syntheses of α melanocyte-stimulating hormone (α-MSH), agouti-related peptide (AGRP) and neuropeptide Y (NPY) through modulating phosphorylation of STAT3 and FOXO1 (Fig. 3). In the adipogenesis pathway, mesenchymal stem cells (MSCs) differentiate into mature adipocytes through various signals including PPARγ as a major regulator (Fig. 4). In the white adipose tissue browning pathway, physical activity induces transdifferentiation of white adipose tissue to beige adipose tissue through various signals including p38 MAPK in the cytosol and Creb, PPARγ, PPARα and VEGFA in the nucleus (Fig. 5). Further studies using comprehensive analysis of mRNAs are needed to determine the targeted molecules that explain the ethnic difference in obesity.

In this study, three circulating miRNAs, miR-125b-1-3p, -20a-5p and -486–5p, which are included in the reported biomarkers for obesity, were associated with the ethnic difference in obesity of Austrian and Japanese men. miR-125b has been shown to target the 3′-UTR of the mRNA of phosphoinositide 3-kinase catalytic subunit δ (PI3KCD), the expression level of which was reported to be decreased in hepatocytes in obese mice [33]. Therefore, PI3KCD is an obesity-related target of miR-125b. PI3K was also suggested by the results of IPA to be a target molecule in leptin signal in obesity (Fig. 3). Thus, in previous studies, miR-125b was associated positively with obesity, while circulating miR-125b was associated negatively with obesity in a previous studies [20] and the present study. The reason for this discrepancy in the results for miRNAs in experiments using cells and blood remains to be clarified in the future. One possible reason for the dissociation between blood levels and cellular levels of miRNAs is the influence on circulating miRNA levels of miRNAs derived from blood cells and vascular cells. In fact, there were strong associations among serum levels of some erythrocyte-derived miRNAs [34], suggesting that some of erythrocyte-derived miRNAs considerably influence their blood levels. On the other hand, circulating miR-20a-5p and miR-486–5p levels were higher in the Austrian group than in the Japanese group. The expression of miR-20a-5p was reported to be induced during adipocyte differentiation from preadipocytes and to be increased in white adipose tissue of obese mice [35]. miR-20a-5p was thought to positively regulate adipocyte differentiation through repressing mRNA of the transducer of ERBB2 (TOB2), although TOB2 was not included in the three pathways related to the ethnic difference in circulating miRNAs in the present study. miR-486 was reported to accelerate preadipocyte proliferation [28], and miR-486–5p level in blood was positively associated with the ethnicity difference in obesity in the present study. However, the target gene(s) of miR-486–5p in relation to obesity remains to be determined in future studies. Thus, both miR-20a-5p and miR-486–5p were positively associated with obesity at both the tissue level and blood level and were significantly different in Austrian and Japanese men in the present study. Therefore, blood levels of miR-20a-5p and miR-486–5p might be useful for detection of a high future risk of obesity in young men in Western countries, although further prospective studies are needed to prove this hypothesis.

The results of linear regression analysis suggest that miR-486–5p levels were associated with miR-125b-1-3p and miR-20a-5p levels, while there was no association between miR-125b-1-3p and miR-20a-5p levels. miR-20a-5p levels showed a strong correlation with miR-486–5p levels, which was independent of miR-125b-1-3p levels in multivariable analysis (*β* = 0.761). This finding appears plausible since both miR-20a-5p and miR-486–5p are involved in adipogenesis as mentioned above [28,35], while miR-125b-1-3p may regulate leptin signal in obesity through affecting PI3K [33].

Although weight and BMI were larger and higher, respectively, in the Austrian group than in the Japanese group, serum leptin and adiponectin levels were comparable in the two groups. In this study, there were no subjects with obesity (BMI of 30 kg/m^2^ or higher) in either the Austrian group or Japanese group. In addition, the percentages of subjects with an upper category of overweight (BMI of 27.5 kg/m^2^ or higher) were 15% in the Austrian group and 10% in the Japanese group. Thus, one possible reason for the above discrepancy is that most of the Austrian and Japanese subjects in this study did not have high BMI. Interestingly, Kuo and Halpern reported that there was no association between BMI and blood adiponectin levels in healthy adults. They speculated that obesity-related changes in adiponectin levels in previous studies were a consequence of obesity-related metabolic disorders [36].

There are limitations of this study. In this study, serum levels of 2565 miRNAs were measured by microarray analysis without amplification; however, only about one third of the total miRNAs showed intensity levels that were high enough for comparison and thus could be analyzed for comparison. Therefore, further studies using measurements with PCR amplification are needed to compare other approximately two thirds of miRNAs in different ethnicities. The subjects of this study were all men, and it is thus necessary to test the ethnic difference in circulating miRNAs in women. Only miRNA expression was evaluated in this study, and simultaneous evaluation of mRNA expression is needed in future studies to clarify molecules that are targeted by miRNAs showing ethnic difference. As candidates of miRNAs explaining the ethnic difference in obesity, we included miRNAs that were shown to differ in obese and non-obese subjects in children as well as adults (Table 1). However, the three miRNAs (miR-125b-1-3p, -20a-5p and -486–5p) that were suggested to explain the ethnic difference in this study were shown in previous studies using data for adults [20,22,28]. There were no significant correlations between BMI and levels of miR-125b-1-3p, -20a-5p and -486–5p (data not shown). The reason for this negative finding may be the limited sample size (n = 40) of our analysis. In addition, subjects with obesity who showed BMI levels of 30 kg/m^2^ or higher were not included in either the Austrian group or Japanese group, although the mean BMI level was significantly higher in the Austrian group than in the Japanese group (25.7 vs. 23.3 kg/m^2^). Therefore, future studies using data from obese Austrian and Japanese participants would be interesting to know ethnic differences in miRNA expression in obese individuals.

In summary, differences in miRNA expression levels in blood were totally analyzed and compared in Austrian and Japanese men. Circulating miR-125b-1-3p, miR-20a-5p and miR-486–5p levels were significantly different between the Austrian and Japanese subjects. By IPA, we provide evidence for an impact of the ethnic differences in the expression of 392 miRNAs on three obesity-related canonical pathways, including leptin signaling in obesity, adipogenesis pathway, and white adipose tissue browning pathway. Thus, miRNAs are thought to partly explain the difference in obesity prevalence in East Asian and Western countries. Future studies are needed to determine the molecules that are regulated by the miRNAs causing the ethnic difference in obesity and the significance of their levels in blood in relation to the pathogenesis of obesity.

## Funding

This study was supported by a Grant-in-Aid for Scientific Research (No. 17H02184) from the 10.13039/501100001691Japan Society for the Promotion of Science (to IW).

## Declaration of competing interest

The authors declare no competing interests.

## CRediT authorship contribution statement

**Ichiro Wakabayashi:** Conceptualization, Data curation, Methodology, Investigation, Visualization, Writing – original draft, Funding acquisition. **Yoko Sotoda:** Data curation, Investigation. **Klaus Groschner:** Investigation, Visualization, Writing – review & editing. **Peter P. Rainer:** Data curation, Investigation. **Harald Sourij:** Conceptualization, Data curation, Investigation, Writing – review & editing.
